# End-stage Renal Disease in Taiwan: A Case–Control Study

**DOI:** 10.2188/jea.JE20080099

**Published:** 2009-07-05

**Authors:** Su-Ying Tsai, Hung-Fu Tseng, Hsiu-Fen Tan, Yu-Shu Chien, Chia-Chu Chang

**Affiliations:** 1 Department of Health Management, I-Shou University, Kaohsiung, Taiwan; 2 Department of Research and Evaluation, Southern California Permanente Medical Group, Kaiser Permanente, Pasadena, CA, USA; 3 Department of Healthcare Administration, Chang Jung Christian University, Tainan, Taiwan; 4 Division of Nephrology, Chang Gung Memorial Hospital, Kaohsiung Medical Center, Chang Gung University College of Medicine, Taiwan; 5 Division of Nephrology, Changhua Christian Hospital, Changhua, Taiwan

**Keywords:** end-stage renal disease, case–control study, risk factors, Taiwan

## Abstract

**Background:**

Taiwan has the highest incidence of end-stage renal disease (ESRD) in the world. The epidemiologic features of ESRD, however, have not been investigated. In this case–control study, we evaluated the risk of ESRD associated with a number of putative risk factors.

**Methods:**

We studied 200 patients among whom ESRD had been newly diagnosed between 1 January 2005 and 31 December 2005; 200 controls were selected from among relatives of patients treated in the general surgery unit. Using a structured questionnaire, we collected information related to socioeconomic factors, history of disease, regular blood or urine screening, lifestyle, environmental exposure, consumption of vitamin supplements, and regular drug use at 5 years before disease onset.

**Results:**

Our primary multivariate risk models indicated that low socioeconomic status was a strong predictor of ESRD (education: odds ratio [OR], 2.78; 95% confidence interval [CI], 1.49–5.19; income: OR, 2.86, 95% CI, 1.48–5.52), even after adjusting for other risk factors. Other significant predictors for ESRD were a history of hypertension (OR, 3.63–3.90), history of diabetes (OR, 3.85–5.50), and regular intake of folk remedies or over-the-counter Chinese herbs (OR, 10.84–12.51). Regular intake of a multivitamin supplement 5 years before diagnosis was associated with a decreased risk of ESRD (OR, 0.12–0.14).

**Conclusions:**

Our findings indicate that low socioeconomic status, history of hypertension, diabetes, and regular use of folk remedies or over-the-counter Chinese herbs were significant risk factors for ESRD, while regular intake of a multivitamin supplement was associated with a decreased risk of ESRD.

## INTRODUCTION

End-stage renal disease (ESRD) is becoming an increasing burden in all regions of the world,^1^ including Taiwan. Data from the US Renal Data System (2007)^2^ indicate that from the year 2000 Taiwan has had the highest incidence and prevalence of ESRD among the countries examined—approximately 400 per million population. Moreover, Taiwan’s 40 000 ESRD patients consume 7% (approximately NT$26 billion) of the national health insurance budget for dialysis treatment, but represent only 0.17% of the population.^3^ The high prevalence of chronic kidney disease and high incidence of ESRD in Taiwan make the study of risk factors for ESRD a high-priority issue for the nation’s health care system. ESRD usually results from slowly progressive kidney damage and is a substantial burden on health care resources. Recent studies reported that awareness and detection of chronic kidney disease (CKD) are low in Taiwan.^4^ Only 8% of patients with stage 3 renal disease in Taiwan are aware of their disease status, as compared to 22% of their counterparts in the United States.^5^ From the perspective of preventive medicine, identification of patients with early renal impairment and those who are at risk for developing kidney diseases may provide an opportunity to decrease the rising incidence of ESRD.

ESRD epidemiologic data, including case–control studies,^6^^–^^11^ have been reported for whites, and they suggest that heavy cigarette smoking,^6^ regular use of home-distilled alcohol,^10^ specific occupational and solvent exposures,^9^^,^^10^ and the regular use of phenacetin or analgesics^7^^,^^8^^,^^10^^,^^11^ are factors strongly associated with chronic renal failure and/or ESRD. Only a few CKD epidemiologic studies have examined ESRD in Asians, and those have been conducted in Thailand^12^ and Japan.^13^^,^^14^ The epidemiologic features of CKD have been investigated in the Taiwanese population^4^^,^^15^^,^^16^; however, these studies were descriptive in nature and equivocal factors that have been associated with ESRD, such as regular screening, lifestyle, environmental exposure, vitamin supplementation, and regular drug use, were not investigated. Previous studies have revealed that aristolochic acid, an ingredient in some Chinese herbal medicines, can lead to kidney failure.^17^^,^^18^ Traditional Chinese herbal medicines are still very popular in Taiwan. Moreover, some people erroneously believe that many of the effective ingredients in Chinese herbs are natural and harmless. Hence, over-the-counter Chinese herb medicines are often purchased and misused. In this study, we specifically examined how the risk of ESRD was affected by the consumption of folk medicines and over-the-counter Chinese herbs used without a prescription. We hypothesized that these factors are independently associated with ESRD. To evaluate this hypothesis, we conducted a case–control study to investigate whether certain factors, such as regular health screening tests, lifestyle, environmental exposure, vitamin supplementation, and regular drug use were associated with the risk of ESRD, after adjustment for socioeconomic factors and variables related to the history of disease.

## METHODS

A retrospective, case–control design was used to examine the association between ESRD and each of the following factors: socioeconomic status (SES), history of medical disease, participation in regular health screening tests, lifestyle, environmental exposures, vitamin supplementation, and drugs used regularly. Attributes in both groups were compared and risk was assessed by using odds ratio for which confidence intervals could be calculated.

### Selection of case and control patients

This case–control study was conducted at 2 tertiary-care medical centers: the Changhua Christian Hospital and the Chang-Gung Memorial Hospital in Taiwan. The subjects were selected and interviewed between 1 January 2005 and 31 December 2005. The cases were dialysis-dependent patients whose new-onset ESRD had been diagnosed between 1 January 2005 and 31 October 2005. Eligibility was limited to patients who were aged from 20 through 64 years at first diagnosis. A total of 263 new-onset cases were identified, and we attempted to interview all eligible patients. After excluding patients who were 65 years old or older at first diagnosis (*n* = 21), patients who had relocated (*n* = 9), and patients who refused to participate (*n* = 33), 200 (76.0%) patients agreed to participate in the interview. The median delay between diagnosis of ESRD and the interview was 2 months.

The controls were relatives of patients who were hospitalized for obstetrical conditions, hemorrhoid surgery, or bone fracture in the 2 hospitals during the study period. Eligibility was limited to subjects aged from 20 through 64 years with no history of any diagnosis relating to kidney disease. Obstetrical conditions, hemorrhoid surgery, and bone fracture are regarded as relatively straightforward diseases or events; hence, it is likely that we avoided selecting control subjects with a familial accumulation of a chronic disease. A total of 281 eligible controls were invited, at which point 200 had agreed to participate. The response rate among controls was 71.2%.

### Data collection

Potential study subjects were initially contacted by invitation. Subsequently, trained interviewers spoke to potential participants face-to face. Before administering the interview, interviewers provided general information regarding the purpose of the study and obtained written informed consent. When possible, the interview was conducted immediately after receipt of the agreement to participate.

Using a structured questionnaire in a face-to-face interview, we collected information about the participant’s demographic characteristics and SES, medical history, participation in regular screening tests of urine and blood, lifestyle habits, environmental exposures, intake of vitamin supplements, and drug usage. Cases were asked to recall exposures 5 years before the diagnosis of ESRD. Although the interviewers were not blinded to the case–control status of participants, they were not informed of the hypotheses under study. Before data collection, the interviewers were carefully trained to conduct a face-to-face interview based on the structured questionnaire, and were instructed in the use of standardized procedures. Each case and control pair was interviewed in person by the same interviewer. The average duration of the interview was 30 minutes. Review by the Institutional Review Board was deemed unnecessary at the time of the study due to the nature of the study. In view of the patients’ rights, the investigators strictly followed ethical rules and obtained informed consent from every participant.

### Exposure variables

Participants’ self-reported demographic data were assessed. SES was determined by level of education and total household monthly income. Level of education was categorized as follows: (1) primary education or no school; (2) secondary education; (3) tertiary education. Total household monthly income was categorized as follows: (1) under US$900; (2) $900 to $1500; (3) above $1500. The participants were asked whether they had been given a diagnosis by a physician of a chronic disease such as hypertension (yes/no) or diabetes (yes/no). Participation in regular preventive screening examinations such as urine testing (yes/no) and blood testing (yes/no) was used as a proxy measure of health awareness. Blood testing was defined as a complete blood count, which is also known as full blood count, full blood exam, or blood panel. Smoking status and alcohol intake were used as lifestyle indices. Alcohol intake included beer consumption, hard liquor consumption, and wine consumption, and was categorized as no habit of alcohol consumption (alcohol consumption only once a week) or habitual alcohol consumption (alcohol consumption more than once a week). Environmental exposure included exposure to certain chemicals such as paint, printing ink, and petrol that might contain polycyclic aromatic hydrocarbons (chemical exposure more than twice a week for at least 6 months). Intake of a vitamin supplement included vitamins B, C, and E, and multivitamins (vitamin supplementation more than once a week). Data on regularly used drugs were collected in the study and comprised the use of antibiotics, steroids, analgesics (including aspirin), and folk remedies or over-the-counter Chinese herbs (drug used more than once a week for at least 6 months). For each factor measured, an effort was made to establish not only the existence of an exposure, but also a rough chronology. Only exposures that had occurred at 5 years before diagnosis of ESRD were considered. For both the case and control, the reference for data collection was the date of diagnosis for the case.

### Statistical analysis

All analyses were performed using Statistical Analysis System (SAS 6.12; SAS Institute, Cary, NC) software. Cases and controls were compared by cross-tabulation analyses and logistic regression for modeling of multiple effects. Odds ratios were used to estimate relative risks. A trend test was performed for ordinal variables whenever a linear trend was suggested by category-specific analyses.

The dependent or outcome variable in the analysis was ESRD. The primary independent or predictor variables of interest were demographic factors (sex, age, education level, monthly income), medical history (hypertension and diabetes), regular screening (urine and blood testing), lifestyle habits (smoking and alcohol intake), environmental exposures (petrol, paint, and printing exposure), use of vitamin supplements (multivitamin, and vitamins C, E, and B), and regular drug usage (antibiotics, steroids, analgesics, and folk remedies or over-the-counter Chinese herbs). Logistic regression was used to estimate the odds ratio (OR) and 95% confidence interval (CI). Univariate analyses were performed between the candidate independent variable and ESRD using simple logistic regression (Tables 1 and 2). A *P* value of less than 0.05 was considered to be statistically significant. Independent variables associated with ESRD with a *P* value of less than or equal to 0.05 in the univariate logistic regression analysis were considered in the multivariate modeling of ESRD, using multiple logistic regression (Table 3). To increase the precision of the estimation without sacrificing validity, we used a backwards procedure to find a “core model” of “important” predictors. Initially all hypothesized risk factors (exposures) were included as covariates in the model, then the factor with the smallest correlation coefficient was dropped, then the factor with the next smallest correlation coefficient was dropped, etc. Thus, each covariate in the core model had an observed effect on ESRD adjusting for all the other predictors in that model. In addition, we tested whether these data fit the model using Hosmer–Lemeshow goodness-of-fit test. Because income and education were closely correlated (test for linear association using the extended Mantel–Haenszel test, *P* < 0.0001), attempting to use income and education in the same model to predict ESRD was inconvenient because they were colinear. We separately examined the association between the 2 factors (education and income) and ESRD in 2 multiple logistic regressions (models 1 and 2).

**Table 1.** Univariate analysis of demographic and socioeconomic variables, medical history, and screening of blood and urine as risk factors for ESRD in Taiwan, 2005

Characteristics	Cases = 200 *n* (%)	Controls = 200 *n* (%)	*P* Value	Crude OR (95% CI)
Demographic factors				
​ Sex				
​ ​ Male	94 (47.0)	63 (31.5)	0.0015	1.92
​ ​ Female	106 (53.0)	137 (68.5)		(1.28–2.89)
​ Age group				
​ ​ 20–40 years	47 (23.5)	80 (40.0)	*<0.0001	1
​ ​ 41–59 years	113 (56.5)	103 (51.5)	test for trend	1.86 (1.19–2.92)
​ ​ 60–64 years	40 (20.0)	17 (8.5)		4.00 (2.04–7.84)

Socioeconomic status				
​ Education				
​ ​ None or primary education	87 (43.5)	26 (13.0)	*<0.0001	1
​ ​ Secondary education	79 (39.5)	68 (34.0)	test for trend	0.34 (0.20–0.59)
​ ​ Tertiary education	34 (17.0)	106 (53.0)		0.09 (0.05–0.17)
​ Monthly income 5 years before diagnosis (US$)				
​ ​ <$900	58 (29.0)	25 (12.5)	*<0.0001	1
​ ​ $900–$1500	65 (32.5)	37 (18.5)	test for trend	0.75 (0.40–1.40)
​ ​ >$1500	77 (38.5)	138 (69.0)		0.24 (0.13–0.41)

Medical history 5 years before diagnosis				
​ Hypertension				
​ ​ Yes	71 (35.5)	23 (11.5)	<0.0001	4.23 (2.51–7.13)
​ ​ No	129 (64.5)	177 (88.5)		
​ Diabetes				
​ ​ Yes	52 (26.0)	9 (4.50)	<0.0001	7.45 (3.54–15.53)
​ ​ No	148 (74.0)	191 (95.5)		

Regular screening 5 years before diagnosis				
​ Urine test				
​ ​ Yes	74 (37.0)	114 (57.0)	<0.000 14	0.44 (0.29–0.66)
​ ​ No	126 (63.0)	86 (43.0)		
​ Blood test				
​ ​ No	73 (36.5)	72 (36.0)	0.9172	1.02 (0.67–1.53)
​ ​ Yes	127 (63.5)	128 (64.0)		

**Table 2.** Estimated effects (ORs and 95% CIs) on ESRD of lifestyle, environmental exposure, vitamin supplementation, and regular drug use 5 years before diagnosis in Taiwan, 2005

Variables		Cases/controls	Crude OR (95% CI)
Lifestyle factors 5 years before diagnosis			
​ Smoking status	No	156/169	0.60 (0.36–1.02)
	Yes	44/29	
	​ cigarettes/day	22.3/20.8	
​ Alcohol intake	No	167/171	0.90 (0.51–1.61)
	Yes	28/26	
	​ years of consumption	20.2/18.7	

Environmental exposure 5 years before diagnosis			
​ Petrol exposure	Yes	23/11	2.23 (1.05–4.71)
	No	177/189	
	​ years of exposure	18.5/14.7	
​ Paint or printing ink exposure	Yes	12/3	4.19 (1.16–15.08)
	No	188/197	
	​ years of exposure	21.7/2	

Vitamin supplementation 5 years before diagnosis			
​ Multivitamin	Yes	16/67	0.17 (0.09–0.31)
	No	184/133	
​ Vitamin C	Yes	10/33	0.26 (0.12–0.55)
	No	190/167	
​ Vitamin E	Yes	7/19	0.34 (0.14–0.84)
	No	193/181	
​ Vitamin B	Yes	30/44	0.62 (0.37–1.04)
	No	170/156	

Regular drug use 5 years before diagnosis			
​ Antibiotics	Yes	24/7	3.75 (1.58–8.94)
	No	176/193	
​ Steroids	Yes	10/2	5.21 (1.12–24.09)
	No	190/198	
​ Analgesics (including aspirin)	Yes	26/16	1.71 (0.89–3.31)
	No	174/184	
​ Folk remedies or over-the-counter	Yes	101/28	6.26 (3.85–10.19)
​ Chinese herb	No	99/172	

**Table 3.** Multivariate odds ratio of ESRD and 95% CI, adjusted for other variables in Taiwan, 2005

Variables	Core Model 1*		Core Model 2*	
	
	Adjusted OR (95% CI)	*P*-value	Adjusted OR (95% CI)	*P*-value
Socioeconomic status				
​ Education ​ ​ (illiterate or primary education vs ​ ​ secondary or tertiary education)	2.78 (1.49–5.19)	0.0012	—	—
​ Monthly income 5 years before diagnosis ​ ​ (<US$900 dollars vs ≥$900 dollars)	—	—	2.86 (1.48–5.52)	0.0017

Medical history 5 years before diagnosis				
​ Hypertension (Yes vs No)	3.90 (2.05–7.42)	<0.0001	3.63 (1.91–6.89)	<0.0001
​ Diabetes (Yes vs No)	3.85 (1.60–9.25)	0.0025	5.50 (2.35–12.86)	<0.0001

Use of vitamin supplement 5 years before diagnosis				
​ Multivitamin (Yes vs No)	0.14 (0.06–0.30)	<0.0001	0.12 (0.05–0.26)	<0.0001

Regular drug use 5 years before diagnosis				
​ Folk remedies or over-the-counter ​ Chinese herbs (Yes vs No)	10.84 (5.77–20.35)	<0.0001	12.51 (6.61–23.68)	<0.0001


Hosmer–Lemeshow test	χ^2^ value	*P*-value	χ^2^ value	*P*-value
	6.0971	0.6364	9.7324	0.2843

*Adjusted for other variables, including age; sex; regular urine testing; petrol, paint, or printing ink exposure; and intake of vitamin C, vitamin E, antibiotics, and steroids.

## RESULTS

The demographic characteristics of cases and controls are summarized in Table 1. There were fewer men among controls (63/200 = 31.5%) than among cases (94/200 = 47.0%) (*P* = 0.0015). Controls were slightly younger than the cases (43.5 years vs 48.4 years). The odds of ESRD cases in males, as compared to females, was 1.92 times higher (*P* = 0.0015). Age, education level, and monthly income showed strong associations with ESRD in univariate analysis, and these trends were highly statistically significant (*P* for trends < 0.0001). The risk gradient steeply increased with age, and ranged progressively from 1.0 to 4.0. The protection gradient steeply increased with education level—progressively ranging from 1.0 to 0.09—and with monthly income, ranging from 1.0 to 0.24. A history of hypertension (OR, 4.23; *P* < 0.0001) and diabetes (OR, 7.45; *P* < 0.0001) were associated with an increased risk for ESRD. A regular urine examination was associated with a decreased risk of ESRD (OR, 0.44; *P* < 0.0001).

The estimated effects on ESRD of lifestyle, environment exposures, vitamin supplementation, and regular drug use are summarized in Table 2. Smoking and alcohol intake were not significantly associated with ESRD. Exposure to petrol (OR, 2.23) and to paint or printing ink (OR, 4.19) moderately increased the risk of ESRD. Participants that reported using multivitamins (OR, 0.17) and vitamins C (OR, 0.26) and E (OR, 0.34) were at lower risk for ESRD. The risk of ESRD increased among those that reported regular use of antibiotics (OR, 3.75), steroids (OR, 5.21), and folk remedies or over-the-counter Chinese herbs (OR, 6.26).

Stepwise backward logistic regression analysis was performed to examine the independent effects of potential factors on ESRD (Table 3). Both of our primary multivariate risk models (models 1 and 2) suggested that low SES (education: OR, 2.78; 95% CI, 1.49–5.19; income: OR, 2.86; 95% CI, 1.48–5.52) was the strongest predictor of ESRD, even after adjusting for other risk variables. Other significant predictors for ESRD were a history of hypertension (OR, 3.63–3.90), diabetes (OR, 3.85–5.50), and the use of folk remedies or over-the-counter Chinese herbs (OR, 10.84–12.51). After controlling for all covariates, a history of multivitamin use was associated with a decreased risk of ESRD (OR, 0.12–0.14). The Hosmer–Lemeshow goodness-of-fit test is a chi-square-based test that is used to assess goodness of fit. In our core model (model 1 and model 2), the chi-square *P* values were 0.6364 and 0.2843, respectively, indicating that these data fit this model.

## DISCUSSION

Taiwan has the highest incidence of ESRD in the world. ESRD reduces quality of life^19^ and can cause clinical depression.^20^ The importance of identifying risk factors for ESRD is evident. In this case–control study, we found that (1) low SES, as measured by either education or income, was a strong independent risk factor for ESRD; (2) both a history of hypertension and diabetes were associated with an increased risk of ESRD; (3) use of a multivitamin supplement was inversely associated with ESRD, suggesting a protective effect; and (4) regular use of folk remedies or over-the-counter Chinese herbs was a strong independent risk factor for ESRD.

Previous studies revealed that low SES may affect health through various mechanisms, including limited access to health services^21^^,^^22^; indeed, studies have indicated that the poor, the uninsured, and African Americans are at increased risk for substandard and delayed care.^23^^–^^29^ In Taiwan, the National Health Insurance (NHI) Program was implemented in 1994 and ensures that care is readily accessible and that patients are free to choose their care providers. With respect to this study, the existence of the NHI likely removes or decreases the influence of some factors, such as limited access to health services due to the lack of health insurance or the financial burden of receiving treatment. However, illiteracy, lower health awareness,^4^ and the greater prevalence of high-risk behavior, including intake of over-the-counter medicine,^27^ may increase the risk for ESRD. In this study (data not shown), when compared to literate participants, illiterate participants had lower awareness of the need for regular urine examination (30.6% vs 53.3%, *P* < 0.0001) and a lower prevalence of multivitamin supplement use (9.0% vs 25.4%, *P* = 0.0003). In addition, illiterate participants had a higher intake of over-the-counter Chinese herbs (45.95% vs 27.18%, *P* = 0.0003) and were more likely to have a history of hypertension (33.3% vs 19.5%, *P* = 0.0035) and diabetes (34.2% vs 7.7%, *P* < 0.0001) than literate participants. Plausible mechanisms for the increased risk of ESRD among the illiterate participants include higher prevalences of hypertension and diabetes; a greater prevalence of high-risk behaviors, including street Chinese herb use; and neglect of regular screening. As mentioned earlier, the existence of the NHI may have reduced the importance of factors related to financial burden; however, income was associated with an increased odds of ESRD (adjusted OR, 2.65; *P* = 0.005) and a significant dose–response relationship was observed (Table 1; *P* for trend <0.0001). This might explain why income in the year before the onset of dialysis was decreased by ill health in patients with ESRD. A US study conducted in Washington, DC and the states of Maryland, Virginia, and West Virginia explored the causal sequence between education, income, and ESRD, and reported that lower education may be a more fundamental cause of ESRD, and that education may be more amenable than income to social intervention.^27^ Further etiologic studies of education, income, and ESRD may shed more light on the causal relationships involved.

The association between hypertension and ESRD has been investigated in previous studies.^30^^–^^34^ Recently, a report based on examining data from the US Renal Data System (2007)^35^ revealed that diabetes is the leading cause of ESRD in the United States, followed by hypertension and glomerulonephritis, and that these 3 conditions accounted for approximately 80% of new ESRD cases treated during 2004. Similarly, an epidemiologic study^16^ in Taiwan that used a nationally representative sample (*n* = 15 271) also revealed that diabetes (adjusted OR, 2.01), hypertension (OR, 1.16), and hyperlipidemia (OR, 1.39) were independent risk factors associated with future development of CKD. In our study, we found an association between ESRD risk and a history of hypertension or diabetes. Furthermore, we also collected information about regular antihypertensive use and separately examined the association between a history of regular antihypertensive use and ESRD. The results indicated that a history of regular antihypertensive use was associated with an increased risk of ESRD (OR, 7.48; 95% CI, 3.62–16.31) after controlling for all covariates except history of hypertension. Studies suggest that even moderate elevation of blood pressure predicted an increased incidence of ESRD,^32^^,^^33^ and that severe, persistent hypertension increased the risk of ESRD.^30^ The findings of this study suggest that most hypertension-related variables are strongly associated with ESRD and demonstrate that diagnosis of hypertension and better blood pressure control are important.

To our knowledge, few epidemiologic studies have explored the effect of multivitamin supplement use on the occurrence of ESRD. In our study, we found that regular intake of multivitamins was inversely associated with ESRD, suggesting a protective effect. Previous studies^36^^–^^38^ revealed that dialysis patients and patients after renal transplantation were prone to vitamin C deficiency. Although studies have suggested that plasma vitamin C levels and multivitamin use predict mortality in dialysis patients,^38^^,^^39^ evidence that multivitamin intake might prevent the occurrence of ESRD is limited. More etiologic studies of the association between multivitamin supplementation and the incidence of ESRD would be worthwhile.

Aristolochic acid, a component of some Chinese herbal medicines, is a well-known cause of severe kidney failure and cancer of the urinary tract system, and Chinese herbal nephropathy can lead to end-stage kidney failure relatively quickly.^17^^,^^18^ A Taiwanese nutrition and health survey^40^ of 1740 adults revealed that Chinese herbal therapy is independently and positively associated with CKD. Many people in Taiwan who consume traditional Chinese medicine and folk remedies habitually use over-the-counter herbal remedies to treat diseases.^41^ Unfortunately, there have been several case reports of Chinese herb nephropathy in Taiwan.^42^^–^^44^ In this study, Chinese herbs are defined as folk remedies or over-the-counter Chinese herbs obtained without prescription. Many herbs in Taiwan are adulterated (up to 23.7%), and the most common adulterants include caffeine, acetaminophen, indomethacin, and hydrochlorothiazide.^45^^,^^46^ These ingredients may be harmful to health. Multivariate analyses revealed an association between the regular use of folk remedies or over-the-counter Chinese herbs and ESRD in our study (OR, 10.84; 95% CI, 5.77–20.35). Among regular users, 51% (65/129) did not know the names of the herbs taken and 73% (94/129) did not know that additional ingredients may have been included. Intake of traditional Chinese herbal medicines is very popular in Taiwan, and some people in Taiwan erroneously believe that many useful ingredients in Chinese herbs are natural and therefore harmless and that this is the reason why the herbs are available over-the-counter. Hence, over-the-counter Chinese herb medicines are often purchased and misused. This may be the principal reason for the high incidence of ESRD in Taiwan.

Case–control studies are susceptible to several biases and limitations. In the present study, exposure measurements were mainly self-reported. ESRD cases may tend to over-report exposures, as compared to controls. In addition, collection of data regarding exposure at 5 years before diagnosis may not be sufficient because it frequently takes longer than 5 years for ESRD to develop. Also, we did not consider the possibility that control subjects from ‘relatives’ may have had diseases other than hypertension, diabetes, and kidney disease; these ignored conditions might have biased the results of this study. The assessment of SES, co-morbidities, and exposure factors using a dichotomized classification could be characterized as simplistic. Moreover, number of cases was relatively small for the large number of stratification analyses that were performed. The confidence intervals of several ORs in this study were large, which reflects the imprecision of estimates due to the relatively small number of patients and controls.

From the perspective of preventive medicine, identification of patients with early renal impairment and those who are at risk for developing kidney diseases may prevent further increases in the incidence of ESRD. Our study demonstrated that low SES, a history of hypertension or diabetes, and regular use of folk remedies or over-the-counter Chinese herbs are strong independent risk factors for ESRD, and that regular intake of a multivitamin supplement is inversely associated with ESRD, which suggests a protective effect.

## References

[1] Schena FP Epidemiology of end-stage renal disease: International comparison of renal replacement therapy . Kidney Int. 2000;57 suppl 74:S39–45 10.1046/j.1523-1755.2000.07407.x

[2] U.S. Renal Data System USRDS 2007 Annual Data Report: Atlas of Chronic Kidney Disease and End-Stage Renal Disease in the United States. Bethesda, MD: National Institutes of Health, National Institute of Diabetes and Digestive and Kidney Diseases; 2007.

[3] Department of Health National Health Insurance Annual Statistical Report Taiwan, ROC, Department of Health, 2004.

[4] Hsu CC , Hwang SJ , Wen CP , Chang HY , Chen T , Shiu RS , High prevalence and low awareness of CKD in Taiwan: a study on the relationship between serum creatinine and awareness from a nationally representative survey . Am J Kidney Dis. 2006;48:727–38 10.1053/j.ajkd.2006.07.018 17059992

[5] Nickolas TL , Frisch GD , Opotowsky AR , Arons R , Radhakrishnan J Awareness of kidney disease in the US population: findings from the National Health and Nutrition Examination Survey (NHANES) 1999 to 2000 . Am J Kidney Dis. 2004;44:185–97 10.1053/j.ajkd.2004.04.023 15264176

[6] Ejerblad E , Fored CM , Lindblad P , Fryzek J , Dickman PW , Elinder CG , Association between smoking and chronic renal failure in a nationwide population-based case-control study . J Am Soc Nephrol. 2004;15(8):2178–85 10.1097/01.ASN.0000135048.35659.10 15284303

[7] Murray TG , Stolley PD , Anthony JC , Schinnar R , Hepler-Smith E , Jeffreys JL Epidemiologic study of regular analgesic use and end-stage renal disease . Arch Intern Med. 1983;143(9):1687–93 10.1001/archinte.143.9.1687 6615090

[8] Morlans M , Laporte JR , Vidal X , Cabeza D , Stolley PD End-stage renal disease and non-narcotic analgesics: a case-control study . Br J Clin Pharmacol. 1990;30(5):717–23 2271370 10.1111/j.1365-2125.1990.tb03841.x PMC1368172

[9] Nuyts GD , Van Vlem E , Thys J , De Leersnijder D , D'Haese PC , Elseviers MM , New occupational risk factors for chronic renal failure . Lancet. 1995;346(8966):7–11 10.1016/S0140-6736(95)92648-8 7603180

[10] Steenland NK , Thun MJ , Ferguson CW , Port FK Occupational and other exposures associated with male end-stage renal disease: a case/control study . Am J Public Health. 1990;80(2):153–7 10.2105/AJPH.80.2.153 2153349 PMC1404604

[11] Fored CM , Ejerblad E , Lindblad P , Fryzek JP , Dickman PW , Signorello LB , Acetaminophen, aspirin, and chronic renal failure . N Engl J Med. 2001;345(25):1801–8 10.1056/NEJMoa010323 11752356

[12] Domrongkitchaiporn S , Sritara P , Kitiyakara C , Stitchantrakul W , Krittaphol V , Lolekha P , Risk factors for development of decreased kidney function in a southeast Asian population: a 12-year cohort study . J Am Soc Nephrol. 2005;16(3):791–9 10.1681/ASN.2004030208 15677313

[13] Imai E , Horio M , Iseki K , Yamagata K , Watanabe T , Hara S , Prevalence of chronic kidney disease (CKD) in the Japanese general population predicted by the MDRD equation modified by a Japanese coefficient . Clin Exp Nephrol. 2007;11(2):156–63 10.1007/s10157-007-0463-x 17593516

[14] Iseki K , Kohagura K , Sakima A , Iseki C , Kinjo K , Ikemiya Y , Changes in the demographics and prevalence of chronic kidney disease in Okinawa, Japan (1993 to 2003) . Hypertens Res. 2007 Jan;30(1):55–62 10.1291/hypres.30.55 17460372

[15] Guh JY , Chen HC , Tsai JF , Chuang LY Herbal therapy is associated with the risk of CKD in adults not using analgesics in Taiwan . Am J Kidney Dis. 2007;49:626–33 10.1053/j.ajkd.2007.02.259 17472844

[16] Kuo HW , Tsai SS , Tiao MM , Yang CY Epidemiology features of CKD in Taiwan . Am J Kidney Dis. 2007;49:46–55 10.1053/j.ajkd.2006.10.007 17185145

[17] Nortier JL , Martinez MC , Schmeiser HH , Arlt VM , Bieler CA , Petein M , Urothelial carcinoma associated with the use of a Chinese herb (Aristolochia fangchi) . N Engl J Med. 2000;342:1686–92 10.1056/NEJM200006083422301 10841870

[18] Nortier JL , Vanherweghem JL Renal interstitial fibrosis and urothelial carcinoma associated with the use of a Chinese herb (Aristolochia fangchi) . Toxicology. 2002;181–182:577–80 10.1016/S0300-483X(02)00486-9 12505369

[19] Blake C , Codd MB , Cassidy A , O’Meara YM Physical function, employment and quality of life in end-stage renal disease . J Nephrol. 2000;13:142–9 10858978

[20] Chilcot J , Wellsted D , Da Silva-Gane M , Farrington K Depression on Dialysis . Nephron Clin Pract. 2008;108:c256–64 10.1159/000124749 18401193

[21] Hayward RA , Shapiro MF , Freeman HE , Corey CR Inequities in health services among insured Americans. Do working-age adults have less access to medical care than the elderly? N Engl J Med. 1988;318:1507–12 3367961 10.1056/NEJM198806093182305

[22] Blendon RJ , Aiken LH , Freeman HE , Corey CR Access to medical care for African American and white Americans . JAMA. 1989;261:278–81 10.1001/jama.261.2.278 2909026

[23] Hostetter TH Research opportunities for reducing racial disparities in kidney disease . Adv Ren Replace Ther. 2004;11:59–65 10.1053/j.arrt.2003.10.009 14730539

[24] Rostand SG , Kirk KA , Rutsky EA , Pate BA Racial differences in the incidence of end stage renal disease . N Engl J Med. 1982;306:1276–9 7040967 10.1056/NEJM198205273062106

[25] Ferguson R , Grim CE , Opgenroth TJ The epidemiology of end-stage renal disease: the six-year South-Central Los Angeles experience. 1980–1985 . Am J Public Health. 1987;77:864–5 10.2105/AJPH.77.7.864 3592045 PMC1647229

[26] Tarver-Carr ME , Powe NR , Eberhardt MS , LaVeist TA , Kington RS , Coresh J , Excess risk of chronic kidney disease among African-American versus white subjects in the United States: a population-based study of potential explanatory factors . J Am Soc Nephrol. 2002;13:2363–70 10.1097/01.ASN.0000026493.18542.6A 12191981

[27] Perneger TV , Whelton PK , Klag MJ Race and end-stage renal disease. Socioeconomic status and access to health care as mediating factors . Arch Intern Med. 1995;155:1201–8 10.1001/archinte.155.11.1201 7763126

[28] Burstin HR , Lipsitz SR , Brennan TA Socioeconomic status and risk for substandard medical care . JAMA. 1992;268:2383–7 10.1001/jama.268.17.2383 1404794

[29] Weissman JS , Stern R , Fielding SL , Epstein AM Delayed access to health care: risk factors, reasons, and consequences . Ann Intern Med. 1991;114:325–31 1899012 10.7326/0003-4819-114-4-325

[30] Perneger TV , Whelton PK , Klag MJ History of hypertension in patients treated for end-stage renal disease . J Hypertens. 1997;15:451–6 10.1097/00004872-199715040-00016 9211180

[31] Walker WG , Neaton JD , Cutler JA , Neuwirth R , Cohen JD Renal function change in hypertensive members of the multiple risk factor intervention trial . JAMA. 1992;268:3085–91 10.1001/jama.268.21.3085 1433739

[32] Klag MJ , Whelton PK , Randall BL , Neaton JD , Brancati FL , Ford CE , Blood pressure and end-stage renal disease in men . N Engl J Med. 1996;334:13–8 10.1056/NEJM199601043340103 7494564

[33] Perry HM Jr , Miller JP , Fornoff JR , Baty JD , Sambhi MP , Rutan G , Early predictors of 15-year end-stage renal disease in hypertensive patients . Hypertension. 1995;25:587–94 7721402 10.1161/01.hyp.25.4.587

[34] Zhang L , Zhang P , Wang F , Zuo L , Zhou Y , Shi Y , Prevalence and factors associated with CKD: a population study from Beijing . Am J Kidney Dis. 2008;51:373–84 10.1053/j.ajkd.2007.11.009 18295053

[35] Centers for Disease Control and Prevention (CDC) Racial differences in trends of end-stage renal disease, by primary diagnosis—United States, 1994–2004 . MMWR Morb Mortal Wkly Rep. 2007;56:253–6 17380108

[36] Morena M , Cristol JP , Bosc JY , Tetta C , Forret G , Leger CL , Convective and diffusive losses of vitamin C during haemodiafiltration session: a contributive factor to oxidative stress in haemodialysis patients . Nephrol Dial Transplant. 2002;17:422–7 10.1093/ndt/17.3.422 11865087

[37] Böhm V , Tiroke K , Schneider S , Sperschneider H , Stein G , Bitsch R Vitamin C status of patients with chronic renal failure, dialysis patients and patients after renal transplantation . Int J Vitam Nutr Res. 1997;67:262–6 9285256

[38] Deicher R , Ziai F , Bieglmayer C , Schillinger M , Hörl WH Low total vitamin C plasma level is a risk factor for cardiovascular morbidity and mortality in hemodialysis patients . J Am Soc Nephrol. 2005;16:1811–8 10.1681/ASN.2004100850 15814831

[39] Andreucci VE , Fissell RB , Bragg-Gresham JL , Ethier J , Greenwood R , Pauly M , Dialysis Outcomes and Practice Patterns Study (DOPPS) data on medications in hemodialysis patients . Am J Kidney Dis. 2004;44 suppl 2:61–7 10.1016/S0272-6386(04)01107-2 15486876

[40] Guh JY , Chen HC , Tsai JF , Chuang LY Herbal therapy is associated with the risk of CKD in adults not using analgesics in Taiwan . Am J Kidney Dis. 2007;49:626–33 10.1053/j.ajkd.2007.02.259 17472844

[41] Kaphle K , Wu LS , Yang NY Herbal medicine research in Taiwan . Evid Based Complement Alternat Med. 2006;3:149–55 10.1093/ecam/nek016 16550238 PMC1375239

[42] Yang CS , Lin CH , Chang SH , Hsu HC Rapidly progressive fibrosing interstitial nephritis associated with Chinese herbal drugs . Am J Kidney Dis. 2000;35:313–8 10.1016/S0272-6386(00)70343-X 10676733

[43] Chang CH , Wang YM , Yang AH , Chiang SS Rapidly progressive interstitial renal fibrosis associated with Chinese herbal medications . Am J Nephrol. 2001;21:441–8 10.1159/000046647 11799260

[44] Hong YT , Fu LS , Chung LH , Hung SC , Huang YT , Chi CS Fanconi's syndrome, interstitial fibrosis and renal failure by aristolochic acid in Chinese herbs . Pediatr Nephrol. 2006;21:577–9 10.1007/s00467-006-0017-6 16520953

[45] Ko RJ Perspective on the adverse reactions from traditional Chinese medicines . J Chin Med Assoc. 2004;67:109–16 15181962

[46] Huang WF , Wen KC , Hsiao ML Adulteration by synthetic therapeutic substances of traditional Chinese medicines in Taiwan . J Clin Pharmacol. 1997;37:344–50 9115061 10.1002/j.1552-4604.1997.tb04312.x

